# Cholinesterase inhibitor to prevent falls in Parkinson’s disease (CHIEF-PD) trial: a phase 3 randomised, double-blind placebo-controlled trial of rivastigmine to prevent falls in Parkinson’s disease

**DOI:** 10.1186/s12883-021-02430-2

**Published:** 2021-10-29

**Authors:** S. Neumann, J. Taylor, A. Bamford, C. Metcalfe, D. M. Gaunt, A. Whone, D. Steeds, S. R. Emmett, W. Hollingworth, Y. Ben-Shlomo, E. J. Henderson

**Affiliations:** 1 University of Bristol, Population Health Sciences, Bristol Medical School, Faculty of Health Sciences, Bristol, UK; 2 North Bristol NHS Trust, Bristol, UK; 3 Royal United Hospitals Bath NHS Foundation Trust, Bath, UK

**Keywords:** Accidental falls, Parkinson disease, Rivastigmine, Cholinesterase inhibitor, Randomized controlled trials

## Abstract

**Background:**

Falls are a common complication of Parkinson’s disease. There is a need for new therapeutic options to target this debilitating aspect of the disease. Cholinergic deficit has been shown to contribute to both gait and cognitive dysfunction seen in the condition. Potential benefits of using cholinesterase inhibitors were shown during a single centre phase 2 trial. The aim of this trial is to evaluate the effectiveness of a cholinesterase inhibitor on fall rate in people with idiopathic Parkinson’s disease.

**Methods:**

This is a multi-centre, double-blind, randomised placebo-controlled trial in 600 people with idiopathic Parkinson’s disease (Hoehn and Yahr stages 1 to 4) with a history of a fall in the past year. Participants will be randomised to two groups, receiving either transdermal rivastigmine or identical placebo for 12 months. The primary outcome is the fall rate over 12 months follow-up. Secondary outcome measures, collected at baseline and 12 months either face-to-face or via remote video/telephone assessments, include gait and balance measures, neuropsychiatric indices, Parkinson’s motor and non-motor symptoms, quality of life and cost-effectiveness.

**Discussion:**

This trial will establish whether cholinesterase inhibitor therapy is effective in preventing falls in Parkinson’s disease. If cost-effective, it will alter current management guidelines by offering a new therapeutic option in this high-risk population.

**Trial registration:**

REC reference: 19/SW/0043.

EudraCT: 2018–003219-23.

ISCRTN: 41639809 (registered 16/04/2019).

ClinicalTrials.gov Identifier: NCT04226248

**Protocol at time of publication:**

Version 7.0, 20th January 2021.

**Supplementary Information:**

The online version contains supplementary material available at 10.1186/s12883-021-02430-2.

## Background

Falls are a common complication of Parkinson’s disease (PD). Prospective studies report that around 61% of people with PD have at least one fall in a year and 39% fall recurrently [1]. Falls are cited as one of the worst aspects of the disease [2], and a major determinant of quality of life, mobility and predictor of life expectancy [3, 4]. The reported median survival in patients who have recurrent falls is around 6 years [5].

Falls can cause injury [6], hospitalisation [7] and fear of further falling [8]. This in turn contributes to social isolation, restricted activity, loss of independence and carer burden [9]. Targeting falls has been identified as a top research priority by people living with Parkinson’s [10].

Current approaches to fall prevention are largely based on physical activity interventions [11]. Whilst exercise training can improve balance and gait and reduce the number of falls [1, 12], the cost-effectiveness and long-term benefit have not been established [13].

To compensate for gait slowing and instability, people with PD need to pay more attention to gait to avoid falling. Cognitive impairment is recognised as a risk factor for falls in Parkinson’s disease and the degree of cognitive impairment in PD is closely related to the incidence of falls [14–16]. Functional imaging has identified two key areas of cholinergic degeneration in the forebrain neocortex and mesencephalic locomotor area [17, 18] that are responsible for cognitive and gait changes, respectively. The loss of cholinergic function leads to cognitive and gait dysfunction. Amelioration of this underlying cholinergic deficiency with cholinesterase inhibitors (ChEis) represents a promising strategy, targeting one of the underlying pathways in the aetiology of falls in PD.

Three small single-centre randomised controlled trials have shown that ChEis may reduce the incidence of falls [14, 19, 20]. Our previous phase 2 placebo-controlled randomised controlled trial suggested that 32-weeks of treatment with oral rivastigmine improved gait variability, walking speed and balance and resulted in a 45% (95% CI 62 to 19%) reduction in fall rate [20].

### Objectives

The CHolinesterase Inhibitors to Prevent Falls in Parkinson’s Disease (CHIEF-PD) trial will compare the fall rates of people with PD treated for 12 months with either transdermal rivastigmine or matched placebo.

## Design and methods

### Design

**Fig. 1:**
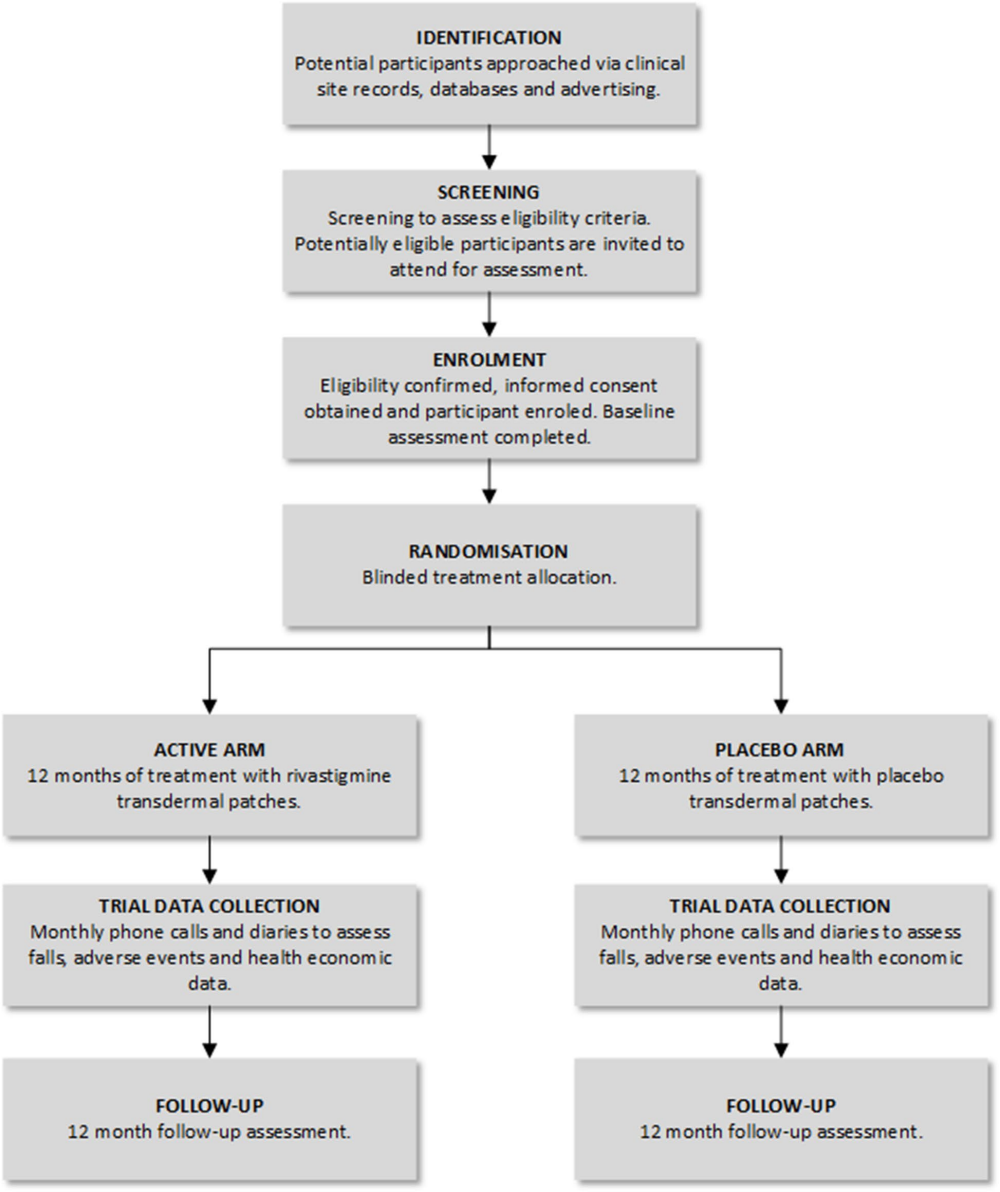
Trial flow chart

CHIEF-PD is a multicentre, placebo-controlled, double-blind, randomised controlled trial using a parallel-arm design (see Fig. 1). In this trial, double blind refers to blinding of the patient, assessor and clinician as well as the research team. Protocol amendments will be brought to the attention of all relevant parties and updated on all relevant registries by the national coordinating team on behalf of the Sponsor.

### Participants and setting

Participants from at least 26 centres in England, Scotland and Wales will be identified by specialist Parkinson’s clinicians. In addition, the trial will be advertised through the Parkinson’s-UK network, and other similar organisations.

Potential participants will be provided with an information booklet giving details of the trial. Eligibility will be assessed according to the criteria in Table 1 and will be confirmed by the site clinician. Written informed consent will be obtained from all participants by the site trial team, and consent will be sought to link their trial data to routine hospital data. Following consent, participants will complete the baseline assessment either face-to-face or using remote video and/or telephone consultation.

**Table 1:** Participants will be eligible if they meet the following criteria

**Inclusion criteria**	
a	Diagnosis of idiopathic Parkinson’s disease.
b	Modified Hoehn and Yahr stage 1 to 4 disease [21].
c	Have experienced a fall in the previous year.
d	Able to walk ≥10 m without aids or assistance.
e	18+ years of age
**Exclusion criteria**	
a	Previous ChEi use during the 12 months prior to enrolment.
b	Hypersensitivity to rivastigmine
c	Dementia diagnosed according to Movement Disorder Society (MDS) criteria [22]
d	Inability to attend or comply with treatment or follow-up scheduling.
e	Non-English-speaking as the cognitive tests are performed in English.
f	Falling ≥4x per day.
g	Unwillingness to use an acceptable method of contraception for the duration of the trial if they are of childbearing potential.
h	Pregnant and/or breast feeding

### Randomisation and blinding

After eligibility has been confirmed and informed consent obtained, participants will be randomly allocated either the active (rivastigmine transdermal patch) or placebo treatment (transdermal patch with no active substance). The randomisation will be stratified by site, and minimised on age (18–64 years versus 65+ years), degree of cognitive impairment (quantified using the Montreal Cognitive Assessment (MoCA score) (1–25 versus 26–30) and number of self-reported falls sustained in the past year (1–4 versus 5+). To avoid the next allocation being predictable, the random allocation ratio is 4:1 in favour of the group that minimises between-group differences on the stratification variables. The randomisation list will be generated by a web-based program which issues a blinded randomisation code that is matched to a transdermal patch treatment, thereby ensuring concealment of allocation (Sealed Envelope Ltd., London, UK). Assessors, clinicians and participants will be blinded to the treatment allocation throughout the trial. Apart from the trial statistician who reports to the Data Monitoring Committee, the research team will only see unblinded analyses of the trial results once assessments are complete and the database has been locked unless there is a clinical indication for unblinding to ensure patient safety in which case the treating physician will contact a central unblinding service.

Blinding will be assessed at the end of the trial using the Schultz questions [23] with the Bang Blinding Index [24]. Both patients and the raters making the monthly phone calls will be assessed.

### Assessment procedures

Participants will undergo assessment at baseline and 12 months. Assessors will receive appropriate training for each assessment undertaken. Assessments may take place face-to-face, or by means of using communication technology such as video calls and/or telephone calls. More than one method of data collection may be used per participant, as determined by the site and participant preference.

Assessments at baseline and 12 months include quantification of fall risk, neuropsychiatric symptoms and cognition, Parkinson’s severity, quality of life, comorbidities and medical and drug history. At baseline and at each titration, an ECG may be collected where the participant has a low heart rate (< 60 beats per minute) or where clinically relevant. The ECG will be collected in clinic, or remotely using the KardiaMobile 6 L device (AliveCor, US).

Quality of Life (QoL) and Cost Effectiveness (CE) will be assessed at months 1, 3, 6, 9 and month 12 of the trial. QoL and CE will be collected by postal questionnaire or over the telephone.

Records of adverse events will be collected throughout the trial via the monthly telephone calls and via spontaneous reporting. Table 2 illustrates the assessments performed at each visit.

**Table 2:**
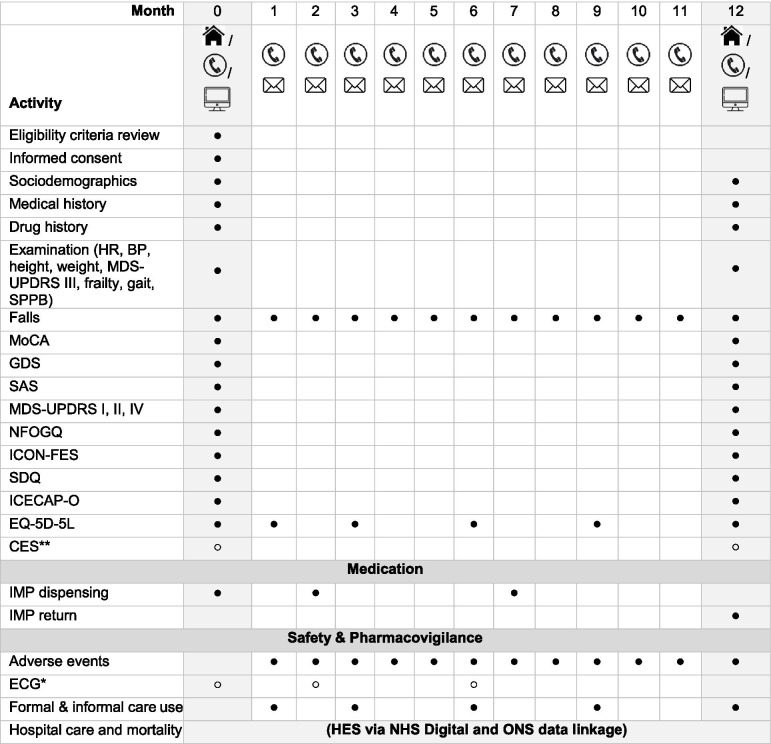
Schedule of assessments and measurement of outcomes

*IMP* Investigational Medicinal Product, *SPPB* Short Performance Physical Battery, *MDS-UPDRS* Movement Disorder Society-Unified Parkinson’s Disease Rating Scale, *NFOGQ* New Freezing of Gait Questionnaire, *ICON-FES* Iconographical Falls Efficacy Scale, *ICECAP-O* ICEpop CAPability measure for Older people, *GDS* Geriatric Depression Scale, *SAS* Starkstein Apathy Scale, *MoCA* Montreal Cognitive Assessment, *CES* Carer Experience Scale, *SDQ* Swallowing Disturbance Questionnaire *ECG as per arrhythmia safety protocol **Completed by carer  Face-to-Face appointment at home or at the hospital,  Remote appointment using video call,  Telephone call,  Postal letter

### Intervention

**Fig. 2:**
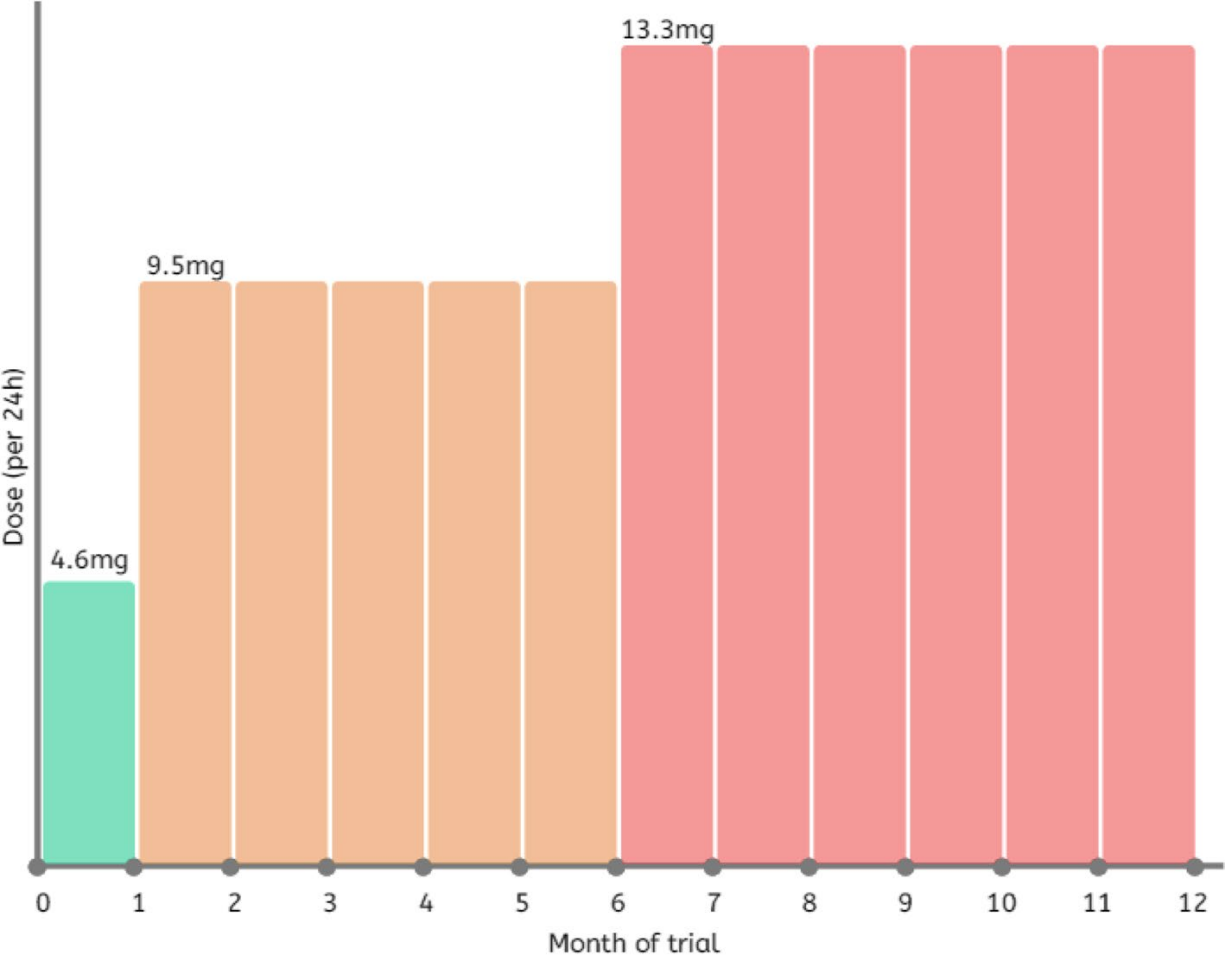
Titration schedule for transdermal patches of rivastigmine or placebo

Participants will receive transdermal rivastigmine or identically matched placebo patches (Luye Pharma, Germany). The transdermal patches will be sent to the participant’s home by Royal Mail or courier following the baseline assessment. Participants will be instructed to apply one patch once a day (having removed the patch applied the previous day). The starting dose will be 4.6 mg/24 h. All participants will up-titrate the dose after 1 month (30 days) to 9.5 mg/24 h, and again at 6 months (180 days) to 13.3 mg/24 h. Participants will remain on 13.3 mg/24 h for the remaining 6 months of the trial. The total treatment duration will be 360 days. The titration schedule is shown in Fig. 2.

If unacceptable side effects are experienced, participants will be instructed to down titrate or cease taking the medication according to clinical advice. These participants will continue to be followed-up for the remaining duration of the trial. Boxes containing the patches will be colour-coded according to the dose to assist with concordance and titration.

### Concordance

Diaries will be used to monitor concordance with the treatment regimen. Participants are required to place a colour-coded sticker in the diary each day which corresponds to the dose of the patch applied. A blue sticker will be available to indicate that no patch was worn in order to differentiate from missing data.

### Primary outcome – fall rate

The primary outcome will be fall rate. Falls will be assessed in accordance with ProFaNE guidance [25] prospectively using self-reported written diaries and monthly phone calls, starting on the day the first transdermal patch is applied. A fall is defined as “unintentionally coming to rest on the ground or other lower surface without overwhelming external force or a major internal event” [25]. At the start of each month of follow-up, the participant will return the previous month’s written diary in pre-paid envelopes to the central research team. The calendar is in a grid format that allows recording of the number of falls and medication.

### Secondary outcomes

The secondary outcomes, in so far as is feasible, utilise outcome measures that are endorsed by the Movement Disorder Society [26].

#### Parkinson’s disease symptoms

Symptoms and stage of Parkinson’s disease will be measured at baseline and 12 months using the Movement Disorder Society’s Unified Parkinson’s Disease Rating Scale (MDS-UPDRS) [21]. The assessment will be made in the practically defined ‘on’ medication state, with participants taking their usual PD, medication and the total score and the score for each sub-scale (part 1–4) will be calculated. Where participants are seen remotely, MDS-UPDRS rigidity and postural stability with retropulsion testing, will not be undertaken.

#### Freezing of gait

Freezing of gait, defined as “an episodic inability to generate effective stepping in the absence of any known cause other than Parkinsonism or high level gait disorders” [27], will be assessed by the New Freezing of Gait Questionnaire [28] which assesses the presence, impact and severity of freezing of gait episodes.

A walk designed to elicit freezing of gait will be assessed which consists of getting up from a chair, walking 5 m in a straight line, then turning 360 degrees in one direction, followed by a 540 degrees turn in the opposite direction, returning to the start position and sitting back down in a chair [29–31]. Freezing of gait will be qualified according to the type of freezing (festination versus tremulous legs versus akinesia), and the location in the walk where the episode occurred (start, straight walk, turning 360 degrees, turning 540 degrees, return walk, sitting down).

#### Frailty and physical performance

Frailty will be assessed by the Survey of Health, Ageing and Retirement in Europe (SHARE) Frailty Instrument [32, 33]. The instrument consists of a questionnaire and measurement of maximum handgrip strength. A Jamar Handgrip or GripX dynamometer will be used to measure handgrip strength (GripX, formerly known as a Camry hand dynamometer).

Physical performance will be assessed by the Short Physical Performance Battery (SPPB, [34, 35]). The SPPB entails balance and gait speed tests. The first balance test asks the participant to stand unassisted whilst placing their feet first side-by- side, then in a semi-tandem position, and finally in a tandem stand, taking each stand for 10 s.

The second balance test asks the participant to stand up repeatedly from a seated position without using their arms. The SPPB gait speed test records the normal walking speed over 4 m in a straight line from a standing start.

The presence of orthostatic hypotension will be determined from blood pressure readings taken upon immediate, 1 min and 3 min of standing following 10 min of supine rest [36]. Where assessments are performed remotely, an Omron automated sphygmomanometer will be sent to the participant. Remote assessment will be supine to seated blood pressure to minimise risk.

Dysphagia will be measured by the Swallowing Disturbance Questionnaire (SDQ, [37]. The questionnaire uses 15 items to address the frequency of swallowing and dysphagia-related difficulties.

The ICEpop CAPability measure for Older people (ICECAP-O), [38, 39], will also be used to measure the broader impact on participant wellbeing. This includes five attributes: attachment, security, role, enjoyment and control.

#### Cognitive and psychometric outcomes

Cognitive ability will be assessed using the Montreal Cognitive Assessment (MoCA, [40]) or MoCA Test Blind whereby the visuospatial/executive and naming sections are omitted and each score is out of 22 which pro-rated to a score out of 30). Where physical disability such as significant tremor prevent the participant from completing the test, it will be adapted by e.g. omitting the visuospatial/executive section with the score out of 25 pro-rated to a score out of 30).

The ability to walk whilst performing a cognitive task, so-called ‘dual tasking’ will be assessed by first recording the participants’ normal walking speed over 10 m, and then asking the participant to repeat the timed walk whilst performing a word fluency test in which the participant is asked to name as many words as possible beginning with a randomly selected letter provided (from: M, H, R, P, D, C, L, A, W, B, or T) [41].

Depressive symptoms will be assessed using the 15-item short geriatric depression scale [42, 43]. Apathy is the most common non-motor symptom of PD [44]. Apathy will be measured using the Starkstein Apathy Scale [45] which consists of 14 self-rated items to address signs of apathy.

Fear of falling will be assessed using the Iconographical 10-item Fall Efficacy Scale (ICON-FES) [46]. This scale uses pictures to describe a range of activities and situations associated with daily living and quantifies the level of concern around the possibility of falling if the activity was undertaken.

#### Health-related quality of life

The EuroQoL 5D-5L health status questionnaire (EQ-5D-5L, [47]) will be administered at baseline, 1, 3, 6, 9 and 12 months using paper forms and/or phone calls. This questionnaire is a generic measure which assesses health related quality of life across 5 domains: mobility, self-care, usual activities, pain/discomfort and anxiety/depression [9], with 5 levels of severity in each domain [47]. An index score anchored at 0 (equivalent to death) and 1 (best health) is derived using a UK value set and can be used to estimate quality-adjusted life-year (QALYs [48]), and allows for the comparison of quality of life between patient groups [49].

#### Mortality

All cause and Parkinson’s disease-related mortality, ascertained as the underlying cause of death, occurring during the 12 months follow-up will be recorded using linked Office of National Statistics mortality data provided by NHS Digital.

#### Health care use

Falls-related NHS hospital visits, admissions and medication changes will be collected each month using patient diaries. At months 1, 3, 6, 9 and 12, patients will complete a questionnaire asking about any community care use, informal care and home adaptations. For participants recruited in England, we will link to data held by NHS Digital on admissions, outpatient and emergency department visits in the 12 months after randomisation. Depending on recruitment numbers and expense, we may also link to routine hospital data in other nations of the UK.

### Pharmacovigilance

Participants will inform the research team if they experience any adverse events (AEs) and be prompted to report these during the monthly phone calls. Serious adverse events will be defined as events that result in death, hospitalisation (except for a pre-existing condition(s) that has not worsened), significant disability or incapacity. AEs will be reported in accordance with the requirements set out by the European Commission Detailed Guidance CT-32011 including the terminology of adverse events and reactions and the assessment of seriousness, causality and expectedness of an event. A phone call will be received at 12.5 months (circa 2 weeks after the follow up visit) to determine whether any withdrawal effects have resulted from medication cessation.

## Carer study

With the participant’s consent, the primary caregiver for each participant will be invited to take part in the CHIEF-PD carer study. For purposes of this trial a carer is defined as an individual who undertakes informal or formal care responsibility for the participant. The aim of the carer study is to ascertain whether cholinesterase inhibitor or placebo treatment has any effect on those caring for participants in the main CHIEF-PD study. The carer will be consented separately from the PD participant. Demographics (gender, age and ethnicity) and the Carer Experience Scale [50] will be collected at baseline and 12 months.

## Sample size

A total of 480 participants with primary outcome data (240 per group) will allow a 25% difference in geometric mean fall rate between the two treatment groups to be detected with 90% power at the two-sided 5% significance level. We previously demonstrated this to be the minimum clinically important difference (MCID) for a falls intervention in Parkinson’s using a Delphi approach [51]. In order to achieve this we have allowed for up to 20% loss to follow-up by setting a recruitment target of 600 participants.

For simplicity, a standard sample size calculation for continuous normally distributed measures was applied to the log transformed falls rates. We have taken into account that the primary analysis will adjust for the baseline measure of fall rates; to be conservative we have assumed that the correlation between baseline and follow-up log-transformed fall rate is 0.58, the lower bound for the 95% confidence interval of this correlation coefficient estimated from our phase II trial data. Log transforming the fall rates from our phase II trial indicates that the control group had a mean of 0.3 (standard deviation 1.2), i.e. a geometric mean of 1.35 falls per month [20]. A 25% reduction in fall rates with treatment corresponds to a − 0.29 reduction on the log scale and hence a mean in the intervention group of 0.012, corresponding to a geometric mean of 1.01 falls per month.

### Statistical analysis

A detailed statistical analysis plan will be written and made publicly available ahead of unblinded data analysis. The primary analysis will follow the intention-to-treat principle as far as possible, by including all participants providing outcome data in the treatment group to which they were randomly allocated.

The log-linear model described in the sample size section for the primary outcome analysis will be elaborated as a mixed Poisson regression model. The number of falls observed for each individual is the outcome variable for this model, with the follow-up period for each participant being incorporated separately, this approach allowing greater flexibility in accommodating variations in follow-up duration. Allocated group, study centre, age at baseline, MoCA cognitive score, and fall history at baseline will be included as covariates. Any individual variation in rate of falls during follow-up will be included in the model using a random effect term with appropriate distribution. The exponential of the coefficient of the allocated group covariate will estimate the treatment effect as a rate ratio. This will be presented with its 95% confidence interval and *p*-value.

This approach will be adapted, by the choice of a suitable regression model, for the analysis of secondary outcomes. Pre-specified subgroup analyses will be specified in the Statistical Analysis Plan.

For the evaluation of safety endpoints, descriptive statistics will describe adverse events for participants who applied at least one patch.

### Economic analysis

Hospital, medications and primary and community care will be costed using national unit costs [52–54]. EQ-5D-5L response at each follow up time point will be converted to utilities using the NICE-recommended UK tariff at the time of analysis. Utility scores will be combined with mortality data to estimate QALYs, controlling for differences in baseline utility scores [55].

The economic analysis will take an intention-to-treat approach with imputation of missing data. In the primary economic analysis, we will estimate the cost-effectiveness of rivastigmine patches over 12 months from the perspective of NHS and social services (to aid comparison with NICE appraisals). Based on the NICE willingness-to-pay thresholds for a QALY we will use net benefit regression to estimate the incremental net benefit (and 95% confidence intervals) [56]. Uncertainty will be explored using cost effectiveness acceptability curves to estimate the probability that rivastigmine is cost-effective at a range of plausible cost-effectiveness thresholds. In secondary analyses we will estimate the cost per fall prevented and expand the perspective of the analysis to include informal care costs, carer quality of life and participant wellbeing. If the intervention has shown sufficient evidence of clinical effectiveness at 12 months, a simple extrapolation model, supplemented with plausible longer-term estimates of costs and effects, will be developed to estimate the cost-effectiveness of transdermal rivastigmine over a patient’s lifetime.

A detailed health economic analysis plan will be developed and made publicly available prior to the analysis.

### Data management

Data collected will be entered onto the CHIEF-PD electronic database and monitored weekly by the national coordinating team. Data will be coded using standard ontologies such as the ICD-10 and the Medical Dictionary for Regulatory Activities (MedDRA). Range checks, missing data and data formats are checked automatically by the electronic database. The data management plan details data security, quality management and access.

Participants consent to sharing their data with the University of Bristol as the data custodian. The University of Bristol ensures that all data is stored confidentially allowing limited access to a subset of the trial team only. Data will remain in the custody of the University of Bristol after the trial in line with the national guidance for Clinical Trials of Investigational Medicinal Products.

Trial data will be published in peer-review publications in line with the NIHR HTA research output policy. Authorship eligibility will be assessed based on the basis NIHR journal’s authorship guidance. The outcome of the trial will also be disseminated to the participants and public at the end of the trial.

## Trial oversight

The trial is overseen by the Trial Steering Group which meets on a biannual basis and comprises external consultees from a clinical, scientific and lay background. The Trial Management Group meets every 2–3 months to discuss the general conduct of the trial. A Data Monitoring Committee consisting of clinical and statistical experts convenes biannually to assess progress, data collection and patient safety. The Data Monitoring Committee along with a trial statistician are unblinded to the treatment allocation. In addition to the internal auditing, the trial will be monitored by the University Hospitals Bristol and Weston NHS Foundation Trust on behalf of the Sponsor.

### Patient and public involvement

The trial design and all patient-facing documents have been designed in collaboration with a Parkinson’s specific patient group. Ongoing collaboration with the group includes advice on trial procedures and dissemination of results.

## Discussion

Falls are common in Parkinson’s and have devastating consequences. A pharmacological strategy to reduce fall risk is a feasible and promising option [20]. This trial will determine whether transdermal treatment with the cholinesterase inhibitor, rivastigmine, can reduce falls in this high-risk group. Rivastigmine is a reversible non-competitive inhibitor of acetylcholinesterase which first received marketing authorisation in 1998 [57] and is currently licensed for use in Alzheimer’s dementia and Parkinson’s dementia [52]. The present trial therefore presents a potential repurposing of a relatively low-cost off-patent medicine. Transdermal patches were selected because of their advantageous side-effect profile, particularly in respect to gastrointestinal symptoms, compared to oral rivastigmine. We will ascertain the effect on motor and non-motor symptoms of PD, including cognition, gait, balance, dysphagia, depression and quality of life. The trial will enable both in-person and remote assessments to be undertaken using video calls and/or telephone calls.

The trial will further establish the cost-effectiveness of the treatment to reduce falls in Parkinson’s Disease, to evaluate whether this may offer an effective, acceptable and affordable intervention repurposing an already established drug.

The trial has been designed in collaboration with patients and uses a primary outcome which is relevant to patients and based on the minimum clinically important difference [51]. Patients have evaluated the methods of trial delivery including the timing of the visits and the acceptability of assessments which we anticipate will lead to high levels of retention and engagement. The primary outcome assessment is informed by best evidence for minimising recall bias and collecting falls data [25]. The secondary outcome measures have, as far as possible, been based on the recommendations from the Movement Disorders Society, to ensure validity in the Parkinson population.

The trial is based on the evidence provided in the phase 2 trial supporting a reduction in fall rate in people with Parkinson’s [20]. The trial will use the gold-standard for providing clinical evidence of efficacy, namely a double-blind, placebo-controlled, randomised controlled trial with minimisation based on age, cognitive ability and number of falls. The inclusion of health economic measures will allow for the evaluation of the treatment for clinical use and recommendation to the National Institute for Health and Care Excellence (NICE).

The trial has been designed with broad and pragmatic eligibility criteria to allow the findings to be as generalisable as possible and reflect the population of people with Parkinson’s who are cared for in specialist clinics. If the results support the use of rivastigmine for falls in Parkinson’s, we will seek to ensure this therapeutic option is incorporated into future management guidelines to ameliorate falls as of the most devastating consequences of the disease.

## Supplementary Information

**Additional file 1.** Model Consent Form.

## Data Availability

Not applicable. The trial results will be published in a peer reviewed journal and made available at the end of the trial. The Investigators will have access to the final trial dataset.

## Abbreviations

CHIEF-PD: Cholinesterase inhibitor to prevent falls in Parkinson’s disease
REC: Research Ethics Committe
EudraCT: European Union Drug Regulating Authorities Clinical Trials Database
ISCRTN: International Standard Randomised Controlled Trial Number
PD: Parkinson’s Disease
ChEis: Cholinesterase inhibitors
MDS: Movement Disorder Society
MoCA: Montreal Cognitive Assessment
ECG: Electrocardiogram
QoL: Quality of Life
CE: Cost Effectiveness
HR: Heart Rate
MDS-UPDRS: Movement Disorder Society Unified Parkinson’s Disease Rating Scale
SPPB: Short Physical Performance Battery
GDS: Geriatric Depression Scale
SAS: Starkstein Apathy Scale
NFOGQ: New Freezing of Gait Questionnaire
ICON-FES: Iconographical Falls Efficacy Scale
SDQ: Swallowing Disturbance Questionnaire
ICECAP-O: ICEpop CAPability measure for Older people
CES: Carer Experience Scale
IMP: Investigational Medicinal Product
ProFaNE: Prevention of Falls Network Europe
SHARE: Survey of Health, Ageing and Retirement in Europe
QALYs: quality-adjusted life-year
NHS: National Health Service
UK: United Kingdom
AEs: Adverse Events
MCID: minimum clinically important difference
NICE: National Institute for Health and Care Excellence
ICD-10: International Classification of Diseases, Tenth Revision
MedDRA: Medical Dictionary for Regulatory Activities
NIHR: National Institute for Health Research
HTA: Health Technology Assessment
MHRA: Medicines and Healthcare Regulatory Agency
