# Supplementary figures and images for: Cholinesterase inhibitor to prevent falls in Parkinson’s disease (CHIEF-PD) trial: a phase 3 randomised, double-blind placebo-controlled trial of rivastigmine to prevent falls in Parkinson’s disease
Source: BMC Neurol. 2021 Oct 29;21:422. doi: 10.1186/s12883-021-02430-2 (PMC8556953; doi:10.1186/s12883-021-02430-2)

**Additional file 1: Model Consent Form**


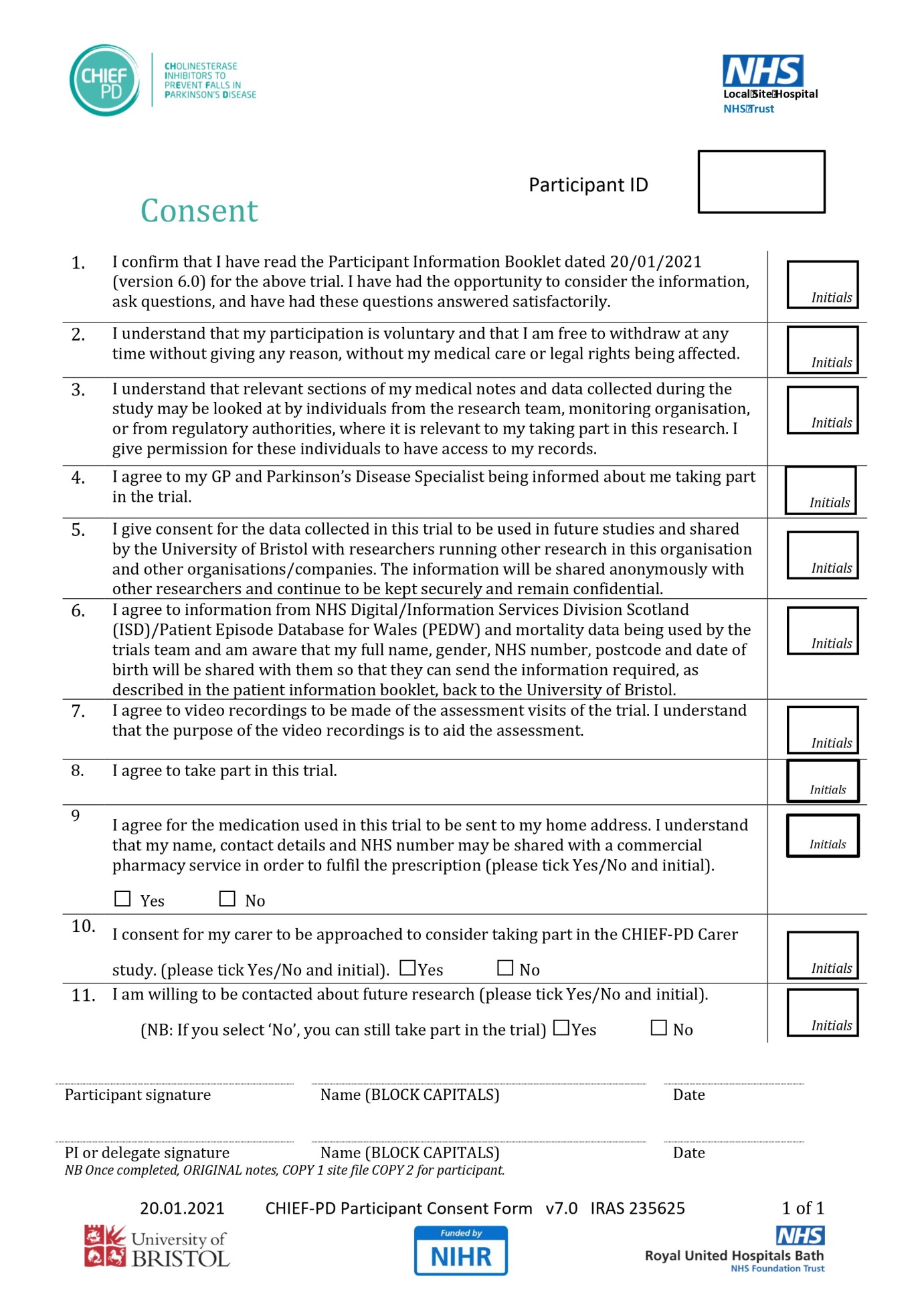

Supplement: Supplementary file 1 — Additional file 1. Model Consent Form. [file 12883_2021_2430_MOESM1_ESM.docx]
